# Physical activity of children and adolescents who use a wheelchair: a systematic review

**DOI:** 10.1186/s12889-023-17201-6

**Published:** 2023-12-11

**Authors:** Selina Seemüller, Franziska Beck, Anne Kerstin Reimers

**Affiliations:** Department of Sport Science and Sport, Friedrich-Alexander-Universität Erlangen-Nürnberg, Erlangen, Germany

**Keywords:** Physical disability, Sports, Physical exercise, Youth

## Abstract

Physical activity has numerous health benefits for people with physical disabilities. Nevertheless, activity levels are often below recommended levels. To promote physical activity among children and adolescents who use a wheelchair as their primary source of mobility, this systematic review explores the physical activity patterns of this group. A systematic search of PubMed, Sports Medicine & Education Index, Web of Science, and SPORTDiscus was performed, included articles were synthesized in terms of duration, intensity, and settings in which physical activity occurred, as well as the physical activity measurement methods. Nine articles were included. The mean overall physical activity level across the included studies was 98 minutes per day (range: 78–115 minutes per day). Two articles analysed the duration of physical activity at different intensities (very light physical activity, light physical activity (LPA), moderate to vigorous physical activity (MVPA) and intensities near to maximum). Within the included articles, both subjective and objective measurement methods were used. Due to the small number of articles, combined with small sample sizes, there is not enough evidence to answer the research questions sufficiently. Nevertheless, the review provides an overview of actual research and clearly shows that the physical activity values are insufficiently researched. There is a need for further research on the scope, types and settings of physical activity in the target group.

## Introduction

Physical activity (PA) “involves people moving, acting and performing in different contexts and settings” [1] and is associated with numerous health benefits for all people [2–4]. In particular, PA improves health, balance, muscle strength, and endurance, counteracts diseases, such as osteoporosis; and increases functional independence, social integration, and life satisfaction [5–7]. Furthermore, PA has important implications for the prevention of obesity, high blood pressure, low fitness levels, and negative clinical outcomes [8, 9]. Engaging in PA and in active lifestyles during childhood and adolescence is crucial, as habits established during this phase frequently persist into adulthood [10].

For children and adolescents with physical disabilities (PD), which the International Classification of Functioning, Disability, and Health defined as impairments, activity limitations, and participation restrictions [11, 12], participating in regular PA is particularly important but challenging: The World Health Organization (WHO) developed PA guidelines for people with disabilities in 2020 [13]. As indicated by these guidelines children and adolescents with PD who are between 5 and 19 years old should perform at least an average of 60 min per day of moderate-to-vigorous-physical-activity (MVPA), especially aerobic activity. Furthermore, muscle and bone strengthening should be incorporated at least three days per week [13, 14]. Additionally, Choi et al. [15] and Sol et al. [16] revealed the positive impact of practice-based interventions on the PA levels of children and adolescents with PD. Consequently, PA is of enormous importance for children and adolescents with PD [17, 18].

Despite this, Bloemen et al. [19] and Sit et al. [20] found that the daily lives of children and adolescents with PD were characterized by high levels of physical inactivity. For example, children and adolescents with PD like Spina Bifida or cerebral palsy are significantly less physically active than their peers without PD [21, 22]. As disabilities can affect mental, physical, and/or developmental impairments [23], children and adolescents with PD face specific barriers: Several reviews have provided an overview of empirical research on PA among youth with PD and identified barriers to PA, such as a lack of appropriate PA programs and insufficient family support [24–26].

As a sub-group of children and adolescents with PD, children and adolescents who use a wheelchair as their primary source of mobility and mode of transport (CAUW) experience various wheelchair-related PA limitations [27]. Like children and adolescents with PD, they often perform less than the aforementioned 60 min of MVPA per day [22, 28]. CAUW face several barriers to participation in leisure and sports activities such as accessibility of playgrounds and sport facilities as well as seasonal effects [16, 29]. Further there are several challenges related to integrating PA into their daily routine due to a lack of suitable opportunities [30].

CAUW’s insufficient PA levels are reflected in their health status. Several studies have shown that wheelchair users have higher body mass index, body fat percentage, and cholesterol levels as well as higher blood glucose concentrations than able bodied individuals [31, 32]. Wheelchair users are also at a higher risk of developing upper extremity overuse injuries, peripheral nerve entrapment, and pressure sores that affect the sacrum and ischial tuberosities [33]. However, PA and sports can help to prevent such injuries [33].

To promote PA in daily life among CAUW, it is important to understand their PA patterns and habits, including how, how often, and where they engage in PA [13], as well as the proportion of CAUW who comply with the WHO PA recommendations. However, to the best of our knowledge, no studies have focused on the PA patterns of CAUW exclusively. To address this gap, the present review aimed to synthesize studies on the PA patterns of CAUW to understand PA duration, intensity levels, types, and contexts [34] and summarize the methods used to measure PA.

## Methods

This systematic review was performed and is reported in accordance with the Preferred Reporting Items for Systematic Reviews and Meta-Analyses (PRISMA) guidelines [35].

### Eligibility criteria

Articles that met the inclusion criteria were included in the present review. Articles that met one or more of the exclusion criteria were excluded. The criteria are listed in Table 1.

**Table 1:** Inclusion and exclusion criteria

	Inclusion Criteria	Exclusion Criteria
Measurement Method	Objective or subjective measurement of PA	
Outcome	PA type, contexts, duration, and patterns	Outcome(s) related to sedentary behavior
Population	• CAUW aged 5–19 years old • Mean age of 5–19 years old • Studies that analyzed participants in the age group from 5 to 19 were also included if this age group was part of a bigger study population	• Participants with non-physical disabilities (e.g., hearing, intellectual, visual impairments) • Participants under 5 years old or over 19 years old
Publication Type	Peer-reviewed journal articles	• Grey literature • Conference abstracts, books, and theses • Publications without peer review
Languages	English, German	• other languages than German or English
Publication Date	2009 to 2022	Before 2009

This review included studies that used different measurement methods to capture a variability of dimensions and aspects of PA in CAUW. Objective measurement methods provide more accurate information regarding the amount and intensity of PA performed by children and adolescents, while subjective measurement methods elicit more background information such as barriers or contributing factors on PA [36, 37].

Further, this review also included interventional and non-interventional studies. The baseline data of the interventional studies offered insights into PA behavior that helped address the objectives of the review.

To ensure the quality of the included studies, only peer-reviewed articles were included in the review. To guarantee the timeliness of the studies, articles from 2009 to 2022 were included because the United Nation Convention on the Rights of Persons with Disabilities came into force and was passed in many countries from 2009 after [38]. Since then, society has been more committed to removing barriers for people with disabilities [39], and it has become easier to participate PA.

### Search strategy

On December 8, 2022, we searched the following electronic databases: PubMed, SPORTDiscus, Sports Medicine & Education Index and Web of Science. Regarding our search strategy, we used a combination of terms related to children and adolescents, PA and wheelchair: ((child* OR adolescen* OR “young people*” OR boys OR girls OR student* OR pupil* OR youth*) AND (“physical activ*” OR sport* OR “physical exercis*” OR “physical inactiv*” OR “physically active” OR “physical fitness” OR “physical condition”) AND (“wheelchair*” OR “walking independen*” OR “walking disab*” OR “walking impairment*” OR “physically restrict*” OR “physically disab*” OR “spina bifida” OR “cerebral palsy” OR “motor impair*” OR “physical impair*” OR “muscular dystrop*” OR “parapleg*” OR “quadripleg*” OR “spinal cord injur*”)).

Three filters were used according to our inclusion criteria to refine the results and obtain the final reference sample for screening. First, the publication date, followed by the publication type was filtered to include journal articles (PubMed: “journal article,” SPORTDiscus: “academic journal,” Sports Medicine & Education Index: “science articles,” and Web of Science: “article”). Third, the publication language was filtered.

### Study selection

After removing all duplicates, two independent reviewers (S.S. and a trained student assistant) enacted a three-step study selection process: (1) title screening, (2) abstract screening, and (3) full-text screening. During each step, all articles that could not be conclusively excluded were kept for further screening in the next step. The researchers were blinded to each other’s decisions. Disagreements regarding final inclusion were resolved through discussions with a third researcher (A.K.R.)

In accordance with recommendations of Briscoe et al. [40] for systematic reviews, we screened the reference lists and citations of the final included articles to identify additional relevant studies. The references were imported into Endnote X9, a reference management software [41].

### Data extraction

The following data were extracted from each article: authors; country; study design; sample characteristics (number of participants, age, gender); study aim/purpose; PA type; PA duration; PA context (e.g., schooldays, weekend days, experimental design, organized sports, daily pattern); and measurement method, instrument, and duration. The data were extracted by one researcher and checked by another (S.S. and a trained student assistant). Disagreements were settled by a third researcher (A.K.R.). Missing data were requested from the study investigators.

### Quality assessment

Two independent reviewers (S.S. and a trained student assistant) rated the quality of the included studies using the Standard Quality Assessment Criteria for Evaluating Primary Research developed by Kmet et al. [42]. Agreement between the raters was measured using the Pearson correlation coefficient. Disagreements were resolved through discussion with a third researcher (A.K.R.).

The QualSyst scoring system by Kmet, Cook [42] assesses quantitative and qualitative research using 14 and 10 items, respectively, that address study design, participant selection methods, random allocation procedures, blinding, outcome measures, sample size, estimation of variance, confounding, reporting of results, and the evidence used to make the conclusion. Each item is scored based on the degree to which it is met (yes = 2, partial = 1, no = 0). If an item is not applicable to a particular study, it is coded with N/A and excluded from the score calculation.

The following equation is applied to estimate the total score for quantitative studies:

$$28-\left(\text{number of n.a.}\times{2}\right)$$

The maximum score that can be obtained for the 14-item evaluation of quantitative studies is 28. The maximum score that can be obtained for the 10-item evaluation of qualitative studies is 20. The risk of bias is evaluated with a summary score (range: 0–1); higher scores indicate better methodological quality.

### Synthesis of the results

Since we expected the studies included in this systematic review to employ a diverse range of research characteristics (e.g., study design, intervention characteristics, contexts, measurements, participant characteristics, and outcome measures), we performed a narrative synthesis of the studies instead of using a meta-analysis to integrate and summarize the studies. Summary tables were created to describe the characteristics of the studies and visualize statistical indicators on the PA duration and type. The studies were grouped according to PA intensity, duration, type, and context and measurement method. PA patterns were analyzed for different categories, which seemed to be helpful in answering the research question.

## Results

### Flow chart

After removing 835 duplicates, title screening was performed on 2,152 potentially relevant articles. Next, abstract screening was performed on 303 articles. Then, full-text screening was performed on 108 articles, and 100 were excluded based on the study aims, statistical analysis, participants, and other reasons. The main reason for exclusion during the full-text screening was the lack of a separate analysis of CAUW. For example, the majority of studies evaluated during that step did not distinguish between wheelchairs and other walking aids.

**Fig. 1:**
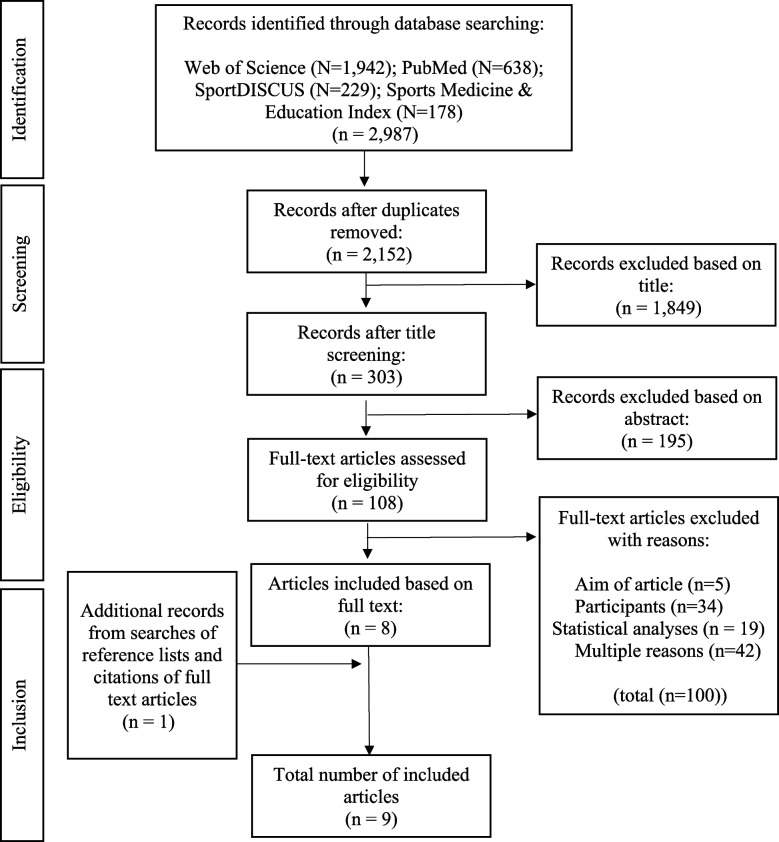
Flow chart

One study was included based on snowball screening. Therefore, nine articles were included in this systematic review (Fig. 1).

### Study characteristics

Table 2 summarizes the characteristics and results of the included studies. Key data, such as authors, country, year of publication, study design, and study aim, were extracted from each article. The sample sizes ranged from 1 [2] to 53 participants [43], with a mean of 20. One article [2] was published in 2010; the rest were published in or after 2017. The let’s ride study was reported in two articles: Bloemen et al. [43] focused on PA duration and intensity in daily life, while Bloemen et al. [19] analyzed the relationships between PA, age, and gender.

**Table 2:** Overview of the study characteristics and results

Author (year); Country	Study Design	Aim	Participants	PA			Measurement		Original results presented in article
			N; Age (years; M±SD); gender	Indicator	Units; Context	Results*	Type; Instrument	total duration of wear time in h	
Bloemen et al. (2019); Netherlands [19]	Observational *Let's ride study*	1. To describe time spent sedentary and physically active of wheelchair-using youth with SB and compare this with typically developing peers; 2. To describe the intensity of daily PA and the compliance to guidelines of PA; 3. To describe the intensity of different types of activities during daily life.	32; 13.7 (3.2); 20 boys, 14 girls	PA	min/ 24 hours; Schoolday	130	Objective; Vitamove, Actiheart	13.2	9% of 13.2 hours of wear time
				PA	min/ 24 hours; weekend	58		10.9	4% of 10.9 hours of wear time
				Wheeling	min/ 24 hours; schoolday	101	Objective; Vitamove, Actiheart	13.2	7% of 13.2 hours of wear time
				Wheeling	min/ 24 hours; weekend	43		10.9	3% of 10.9 hours of wear time
				Walking	min/ 24 hours; schoolday	0		13.2	0% of 13.2 hours of wear time
				Walking	min/ 24 hours; weekend	0		10.9	0% of 10.9 hours of wear time
				Running	min/ 24 hours; schoolday	0		13.2	0% of 13.2 hours of wear time
				Running	min/ 24 hours; weekend	0		10.9	0% of 10.9 hours of wear time
				(Hand)-biking	min/ 24 hours; schoolday	0		13.2	0% of 13.2 hours of wear time
				(Hand)-biking	min/ 24 hours; weekend	0		10.9	0% of 10.9 hours of wear time
				Non-cycling movement	min/ 24 hours; schoolday	0		13.2	0% of 13.2 hours of wear time
				Non-cycling movement	min/ 24 hours; weekend	0		10.9	0% of 10.9 hours of wear time
				Very Light PA (0-30%)	min/ 24 hours; schoolday	1088	Objective; Vitamove, Actiheart	12.7	575 min of 761 min wear time
					min/ 24 hours; weekend	1146		10.5	500 min of 628 min wear time
				LPA (30-40%)	min/ 24 hours; schoolday	208		12.7	110 min of 761 min wear time
					min/ 24 hours; weekend	128		10.5	56 min of 628 min wear time
				MPA (40-60%)	min/ 24 hours; schoolday	104		12.7	55 min of 761 min wear time
					min/ 24 hours; weekend	46		10.5	20 min of 628 min wear time
				VPA (60-90%)	min/ 24 hours; schoolday	19		12.7	10min of 761 min wear time
					min/ 24 hours; weekend	2		10.5	1min of 628 min wear time
				Near to max. (>90%)	min/ 24 hours; schoolday	0		12.7	0min of 761 min wear time
					min/ 24 hours; weekend	0		10.5	0min of 628 min wear time
				Very Light PA; 0-30 %	Experimental design	lying, sitting, non-cycling	Objective; Vitamove, Actiheart	n.a	n.a.
				LPA; 30-40%	Experimental design	Standing, wheeling, (hand)biking		n.a	n.a.
				MPA; 40-60%	Experimental design	Walking		n.a	n.a.
Bloemen et al. (2020); Netherlands [43]	Exploratory *Let's ride study*	To analyse the associations between PA and VO2peak, age, sex and Hoffer classification in young wheelchair-users with SB.	34; 13.7 (3.2); 20 boys, 14 girls	PA	min/ 24 hours; schoolday	128	Objective; Vitamove	58	8.9% of 2.4 days wear time
					min/ 24 hours; weekend	58			4% of 2.4 days wear time
			36; 13.5 (3.6); 21 boys, 15 girls	MVPA	min/ 24 hours; schoolday	130	Actiheart	65	9% of 2.7 days wear time
					min/ 24 hours; weekend	58			4% of 2.7 days wear time
			59; n.a.; boys	PA	min/ 24 hours; schoolday	115	Vita Move, Hoffer classification	58	8% of 2.4 days wear time
					min/ 24 hours; weekend	58			4% of 2.4 days wear time
			Girls	PA	min/ 24 hours; schoolday	173			2% of 2.4 days wear time
					min/ 24 hours; weekend	86			6% of 2.4 days wear time
			64; n.a.; Boys	MVPA	min/ 24 hours; schoolday	144	Actiheart; Hoffer classification	65	10% of 2.7 days wear time
					min/ 24 hours; weekend	58			4% of 2.7 days wear time
			Girls	MVPA	min/ 24 hours; schoolday	101			7% of 2.7 days wear time
					min/ 24 hours; weekend	72			5% of 2.7 days wear time
			n.a.; n.a.; n.a.	PA	n.a.; schoolday	PA decreases with increasing age of (-0.369)	Vita Move, Spearmans rank correlation (PA & Age)	n.a.	n.a.
			n.a.; n.a.; n.a.		n.a.; weekend	PA decreases with increasing age of (-0.286)			
			n.a.; n.a.; n.a.	MVPA	n.a.; schoolday	MVPA decreases with increasing age of (-0.311)	Actiheart, Spearmans rank correlation (PA & Age)	n.a.	n.a
			n.a.; n.a.; n.a.		n.a.; weekend	MVPA decreases with increasing age of (-0.512)			
Buffart et al. (2010); Netherlands [2]	Intervention	To present the design and procedures of the ALSP intervention.	1; 17; Boy	energy expanditure	kJ/kg/day; experimental design	43	Subjective; Questionnaire: PASIPD	n.a.	n.a.
Kanagasabai et al. (2018); New Zealand [44]	Interview	To develop a deeper understanding of the leisure participation experiences of children with movement impairments to be better able to inform families, therapists and community service providers about ways to improve engagement in leisure activities of children with movement impairments for their overall development and well-being.	9; 8.4; 7 boys, 2 girls	Organized sports	Type of sports (amount of all participants)	Archery (1) climbing (1) Rugby (1)	Subjective; Interview	n.a.	n.a.
Lauruschkus et al. (2017); Sweden [45]	Intervention	To explore how parents of children experience their child’s participation in physical activities and to identify facilitators and barriers for being physically active.	25 parents of 16 children (8-11 years); n.a.; 10 male, 15 female	PA	n.a.; n.a.	More passiv on school, more active at home	Subjective; Interview	n.a.	n.a.
Sol et al. (2019); Netherlands [46]	Mixed-method	To develop a questionnaire to assess confidence in wheelchair mobility in Dutch youth (WheelConMobility Dutch Youth).	8; 12 (8-17); 5 boys, 3 girls	Organized Sports	Number of participant in sport	6	Objective; Questionnaire	n.a.	n.a.
Sol et al. (2022); Netherlands [16]	Intervention	To evaluate the effects of a combination of wheelchair mobility skills training and exercise training on physical activity (PA), WMS, confidence in wheelchair mobility, and physical fitness.	35; 12.8 (3.1); n.a.	PA	min/ 24 hours; schoolday, weekend	94	Objective; Activ8 activity monitor	8	6,5% of 8 hours wear time / day
van der Geest et al. (2020); Netherlands [47]	Cross-sectional	To evaluate the validity of home measurements of upper extremity accelerometry.	2; n.a.; boys	Organized Sport	min/ 24 hours; n.a.	60	Subjective; PA diary	12.5	60min Sports at school
									60min Physiotherapy
				PA	min/ 24 hours; n.a.	120	Subjective; PA diary		120min Playiing WII with friends
Walker et al. (2020); USA [48]	Photovoice	To examine barriers and facilitators to physical activity in rural youth with CP from the perspectives of children and parents and to elicit in-depth responses.	7 childen; (14.1 - 21); 4 boys, 3 girls	Organized sport	n.a.; n.a.		Subjective; Interview	n.a.	n.a.
			8 parents; n.a.; 8 female	Organized sport	Type of sports (amount of all participants)	Climbing (1) Basketball (1)	n.a.	n.a.	n.a.

*Note: Values are calculated based on the raw data.

*PA *Physical activity, *LPA *Light physical activity, *MPA *Moderate physical activity, *MVPA *Moderate to vigorous physical activity, *n.a *not applicable

### Data synthesis

Table 3 presents the synthesis of the PA characteristics of the included studies. The majority of included articles presented non-interventional studies (*n* = 6; 67%), such as observational, mixed-method, cross-sectional and exploratory designs as well as interview studies. Two studies measured PA using objective measurement method, namely, accelerometers [16, 19, 43]. One study used objective and subjective measurement methods [2]. Four studies used the following subjective measurement methods: interviews ([44], photovoice [48], subjective questionnaires [2] and PA diaries [47]. Objective measurement methods revealed higher PA values than subjective ones. Further, age structures were equally distributed across all measurement methods.

**Table 3:** Data synthesis

		Number of Articles (% of all)	Number of Participants (% of all)	Main Outcome		Authors (Year)
Type of Day	Schoolday	3 (33.3)	120 (66.7)	Duration in min/day: mean value (mean range)	117 (108–132)	Bloemen et al., 2019 [19], Bloemen et al., 2020 [43]; Sol et al., 2022 [16]
	Weekend day	3 (33.3)	120 (66.7)	Duration in min/day: mean value (mean range)	71 (70–79)	Bloemen et al., 2019 [19], 2020; Sol et al., 2022 [16]
PA Type/ context	Overall PA	5 (55.5)	147 (81.7)	Duration in min/day: mean value (mean range)	98 (78–115)	Bloemen et al., 2019 [19], Bloemen et al., 2020 [43]; Sol et al., 2022 [16]; van der Geest et al., 2020 [47]
	Energy expenditure	1 (11.1)	1 (0.6)	N/A		Buffart et al., 2010 [2]
	Wheeling	1 (11.1)	32 (17.8)	Duration in min/day: mean value (mean range)	72 (43 –101)	Bloemen et al., 2019 [19]
	Organized sports	3 (33.3)	32 (17.8)	Type of sports	Archery, climbing, rugby, basketbal	Kanagasabai et al., 2018 [44]; Sol et al., 2019 [46]; Walker et al., 2020 [48]
PA Intensity	LPA	1 (11.1)	32 (17.8)	Duration in min/day: mean value (mean range median)	168 (128–208)	Bloemen et al., 2019 [19]
	MPA	1 (11.1)	32 (17.8)	Duration in min/day: mean value (mean range median)	75 (46–104)	Bloemen et al., 2019 [19]
	MVPA	1 (11.1)	53 (29.4)	Duration in min/day: mean value (mean range median)	94 (58–144)	Bloemen et al., 2020 [44]
	VPA	1 (11.1)	32 (17.8)	Duration in min/day: mean value (mean range median)	11 (2–19)	Bloemen et al., 2019 [19]
	Near to maximum	1 (11.1)	32 (17.8)	Duration in min/day: mean value (mean range median)	0 (0–0)	Bloemen et al., 2019 [19]

*PA *Physical activity, *LPA *Light physical activity, *MPA *Moderate physical activity, *MVPA *Moderate to vigorous physical activity, *VPA *Vigorous physical activity, *N/A *Not applicable

The nine included studies examined PA components of 180 CAUW. Regarding the PA context, [16, 19, 43] revealed that CAUW moved significantly more on schooldays (average: 117 min per day) than on weekend days (average: 70 min per day) [19, 43]. examined PA based on the following intensity levels: very light PA: 0–30%, light PA (LPA): 30–40%, moderate PA (MPA): 40–60%, vigorous PA (VPA): 60–90%, and near to maximum intensities: >90%. The findings indicated that increased PA intensity led to reduced PA duration.

Further, regarding the PA durations across all intensities CAUW revealed high PA levels with a mean of 98 min per day (range: 78–115 min per day) [16, 19, 43, 47]. Daily activities such as wheeling revealed light intensities and a mean of 72 min and a range of 43 − 101 min per day [19].

### Quality assessment results

Table 4 shows the methodological quality assessment results for the quantitative studies (*n* = 4). The QualSyst results obtained by both raters were medium to good (mean = 0.78, SD = 0.12, range 0.73–0.95). Additionally, the Pearson correlation coefficient between the raters was high (0.872; [49].

**Table 4:** QualSyst results for the quantitative studies

		Studies fulfilling the criteria n				Detailed quality assessment Yes = 2; Particular = 1; No = 0; n.a.			
**No.**	**Item**	**Yes**	**Partial**	**No**	**N/A**	**Bloemen et al. (2019)** [19]	**Bloemen et al. (2020)** [43]	**Sol et al. (2019)** [46]	**Sol et al. (2022) **[16]
1	Question /objective is sufficiently described?	4	0	0	0	2	2	2	2
2	Study design is evident and appropriate?	3	1	0	0	1	2	2	2
3	Method of subject/comparison group selection or source of information/input variables is described and appropriate?	4	0	0	0	2	2	2	2
4	Subject (and comparison group, if applicable) characteristics are sufficiently described?	3	1	0	0	2	1	2	2
5	If interventional and random allocation was possible, was it described?	0	2	2	0	1	n.a.	n.a.	1
6	If interventional and blinding of investigators was possible, was it reported?	0	2	0	2	n.a.	n.a.	n.a.	0
7	If interventional and blinding of subjects was possible, was it reported?	0	0	1	3	n.a.	n.a.	0	0
8	Outcome(s) and (if applicable) exposure measure(s) is/are well defined and robust to measurement/misclassification bias? Means of assessment are reported?	2	2	0	0	2	2	1	1
9	Sample size is appropriate?	3	1	0	0	2	2	1	2
10	Analytic methods are described/justified and appropriate?	4	0	0	0	2	2	2	2
11	Some estimate of variance is reported for the main results?	1	2	1	0	1	2	1	1
12	Controlled for confounding?	0	0	2	2	0			0
13	Results reported in sufficient detail?	4	0	0	0	2	2	2	2
14	Conclusions supported by the results?	2	2	0	0	1	2	1	2
Summary score (total sum / total possible sum)						0.75	0.95	0.73	0.68

Table 5 shows the methodological quality assessment results for qualitative studies (*n* = 5). There was a medium pearson correlation coefficient between the raters (*r* = 0.553, *p* = 0.334; [49]. Like the quantitative studies, the QualSyst results obtained by both raters for the qualitative studies were medium to good (mean = 0.76, SD = 0.07, range = 0.65–0.85).

**Table 5:** QualSyst results for the qualitative studies

		Studies fulfilling the criteria n				Detailed quality assessment Yes = 2; Particular = 1; No = 0; n.a.				
**No.**	**Item**		**Yes**	**Partial**	**No**	**Buffart et al., (2010) **[2]	**Kanagasabai et al., 2018** [44]	**Lauruschkus et al., 2017** [45]	**van der Geest et al., (2020)** [47]	**Walker et al., (2020)** [48]
1	Question / objective clearly described?		5	0	0	2	2	2	2	2
2	Design evident and appropriate to answer study question?		4	1	0	2	2	2	1	2
3	Context for the study is clear?		5	0	0	2	2	2	2	2
4	Connection to a theoretical framework / wider body of knowledge?		3	2	0	2	2	1	1	2
5	Sampling strategy described, relevant and justified?		3	2	0	1	2	1	2	2
6	Data collection methods clearly described and systematic?		3	2	0	1	2	1	2	2
7	Data analysis clearly described, complete and systematic?		4	1	0	1	2	2	2	2
8	Use of verification procedure(s) to establish credibility of the study?		0	0	5	0	0	0	0	0
9	Conclusions supported by the results?		3	1	1	1	2	2	2	0
10	Reflexivity of the account?		2	3	0	1	1	2	1	2
Summary score (total sum / total possible sum)						0.65	0.85	0.75	0.75	0.8

## Discussion

The objective of the present systematic review was to synthesize peer-reviewed studies on the PA patterns of CAUW to understand PA duration, intensity levels, types, and contexts and summarize the methods used to measure PA. Nine articles that investigated PA among CAUW were included in the review. A detailed analysis of the study characteristics, including study design; participant characteristics; PA context, duration, and type; and measurement instrument and duration, was conducted. Furthermore, various PA characteristics were summarized to provide insights into the habitual PA of CAUW.

### Actuality of articles

The majority of the articles were published between 2017 and 2022 (*n* = 8). This observation is in line with the increase in the number of studies conducted on the broader topic of PA among children with disabilities over the last decade [50, 51]. Within society, there is a growing attention for inclusion and the dismantling of barriers for people with disabilities [52]. The number of articles could be due to the increasing importance and awareness of inclusion in society.

### Number of articles

Nonetheless, the small number of articles included in this review is indicative of a pressing need for further research on PA among CAUW. Moreover, due to the small number of articles (*n* = 9) and the small sample sizes (range_N_ = 1–53 participants), the results of the present review cannot be considered reliable [53]. Nevertheless, the review provides an overview of actual research and clearly shows that the PA values of CAUWs are insufficiently researched. Several studies included wheelchair users in samples of children and adolescents with disabilities but did not analyze their PA separately [54, 55], perhaps due to difficulties related to recruiting CAUW only. Due to the various PA-related barriers and needs of CAUW, it is necessary to report meaningful results separately for this group [27, 56].

### Levels of PA

Nevertheless, some findings of the review should be discussed. For example, the review indicated that PA intensity impacts PA duration. Bloemen et al. [43] identified a mean of 94 min of MVPA per day. Four studies revealed a mean of 98 min (range: 78–115) of overall PA across all intensities per day [16, 19, 43, 45, 47]. Bloemen et al.  [19] identified a mean of 72 min of habitual PA (range: 43–101), such as wheeling, per day and showed that the WHO-recommended level of 60 min of MVPA [13] was met.

### Data synthesis

To compare the results of the included articles, the data were converted into minutes per day (24 h). This approach may have resulted in a methodological weakness because time factors, such as sleeping, could not be taken into account, leading to PA levels that might have been too high. To address this issue, we included the original study results in Table 1. Furthermore, the data obtained from accelerometers were estimates. Since they often do not meet the gold standard for free-living PA measurements, they should not be interpreted as accurate PA values [57].

### Measurement duration

Meanwhile, most participants in Bloemen et al. [19] study took part in sports one to three times per week. If at least one of these activities was within the short measurement period of up to three days, that could explain the high PA levels and MVPA results. To avoid such issues and put snapshots of PA into perspective, Montoye, et al. [58] recommended that objective measurement periods should have an average duration of at least one week with a wear time of 10 h per day.

### Measurement methods

Regarding measurement methods, the advantages, disadvantages, and methodological features of the instruments affected how they were used in the included studies. For example, Sol et al. [16], Bloemen et al. [19, 43] used accelerometers to capture PA duration and intensity, while Kanagasabi et al. [44] and Lauruschkus et al. [45] employed interviews to focus on PA type. Accelerometers more accurately capture quantitative aspects of PA, such as duration, frequency, and intensity. In contrast, interviews more accurately capture qualitative aspects of PA, such as type, and have limited validity with regard to duration and intensity [59]. Nonetheless, given the limited data available on PA among CAUW, future research should focus on the quantitative and qualitative aspects of PA. For example, a comprehensive overview of PA among CAUW could be obtained through a mixed-method approach, such as an ecological momentary assessment methodology in which accelerometers and survey methods are used simultaneously [60].

### PA and health

The studies included in the present review did not offer clear findings on the relationship between PA and health. Since, as previously mentioned, children and adolescents should engage in at least a moderate level of PA to achieve health benefits [61], overall PA is not a very meaningful indicator because it does not address intensity or context. Furthermore, wheeling, which has been classified as LPA, has little effect on health [19]. Moreover, the lack of data on PA intensity and type in most of the included studies hindered the examination of the results in a health context [62].

Regarding the PA context, Sol et al. [16] and Bloemen et al. [19, 43] found higher PA levels on schooldays than on weekend days. Siegmund et al. [63, 64] found the same result in studies of children and adolescents who did not use wheelchairs. Regarding PA type, Doorley et al. [65] and Kjønniksen et al. [66] revealed that school sports and active travel to school (e.g. by bike or by walking) could increase participants’ PA levels on schooldays. Consequently, future studies should explore the promotion of PA on weekend days, such as weekend sports activities at sports clubs.

To obtain valid insights into health-related PA among CAUW, future studies should collect data from a larger number of participants over a longer time period. Therefore, the difference of PA in various weekdays should be noted to exclude snapshots of the overall PA (e.g. when CAUW have club training once a week and this is when the PA was measured). Furthermore, PA intensity should be considered in relation to PA duration and type. In addition, future research should examine whether CAUW meet PA WHO recommendations and examine the factors that affect their ability to meet the WHO recommendations.

### Type of research

Qualitative studies of PA types and contexts, such as organized sports and energy expenditure, are less suitable for obtaining reliable data on PA intensity and frequency per week than objective measurement methods [44, 45, 47, 48]. For example, it was unclear whether participants engaged in organized sports more than once a week or if the data merely reflected a snapshot of a single day. Future studies should examine PA types that have not yet been considered, such as active travel. To answer the present review’s research question concerning PA among CAUW, it is necessary to identify different factors and consider PA type and duration together.

### Practical implications

Existing barriers such as accessibility of built environments as well as difficult possibilities to participate in sport groups influence PA-levels of CAUW [45, 48]. Therefore, in order to increase PA-levels, it is of enormous importance to identify and reduce existing barriers. Further research should also focus on barriers and facilitators regarding PA in CAUW.

### Strengths and limitations

The main strength of the present review was the inclusion of studies that objectively and subjectively measured PA, enabling the examination of a variety of PA patterns and variables (e.g., frequency, intensity, time, type). Furthermore, the systematic literature search employed several electronic databases and a comprehensive list of search strings. In addition, the reference lists of all included articles were manually checked to obtain other relevant studies. The search strategy was broad enough to identify relevant studies, including ones that did not make overall PA analysis their main objective. Unlike reviews in the broader PD research field [24, 67], the present review only included studies that exclusively focused on CAUW to clearly connect PA patterns to wheelchair use. Research on multiple impairments cannot guarantee that the observed behavior is not due to a secondary impairment.

Regarding limitations, the variety of study designs made it difficult to compare and synthesize the results of the included studies. To address this issue, the results were converted into minutes per day (24 h). However, PA levels might have been too high because time factors, such as sleeping, could not be taken into account. In addition, only nine studies with small sample sizes were included in this review due to the lack of sufficient studies in the field. The review’s findings cannot be generalized or considered representative due to this small number of studies and sample sizes.

## Conclusion

This systematic review yielded inconclusive results regarding PA among CAUW. The nine included articles examined PA components related to a total of 180 CAUW. The data synthesis revealed connections between PA intensity, duration, type, and context. The classification of wheeling as an LPA can be useful for further research. There is a need for high-quality studies of PA among CAUW with larger sample sizes as well as studies of different daily PA contexts.

Based on the present review, no valid conclusions can be made. To gain a deeper understanding of the PA patterns and needs of CAUW and the barriers they face, a combination of quantitative and qualitative measures could be used to simultaneously collect valid data on PA domains, such as duration, intensity, type, and context.

## Data Availability

All data generated or analyzed during this study are included in this published article.
